# Peptidome Analysis of Pancreatic Tissue Derived from T1DM Mice: Insights into the Pathogenesis and Clinical Treatments of T1DM

**DOI:** 10.1155/2021/9987042

**Published:** 2021-05-21

**Authors:** Fan Zhang, Meiyun Zhou, Shuangshuang Li, Jinhua Gu, Yuanyuan Qian, Sisi He, Li Hong, Linlin Sun, Xiaohua Zhang, Weigang Ji

**Affiliations:** ^1^Department of Pediatrics, Affiliated Maternity and Child Health Care Hospital of Nantong University, Nantong, Jiangsu, China; ^2^Department of Nantong Institute of Genetics and Reproductive Medicine, Affiliated Maternity and Child Health Care Hospital of Nantong University, Nantong, Jiangsu, China; ^3^Department of Clinical Pharmacy, Affiliated Maternity and Child Health Care Hospital of Nantong University, Nantong, Jiangsu, China

## Abstract

Bioactive peptides attract growing concerns for their participation in multiple biological processes. Their roles in the pathogenesis of type 1 diabetes mellitus remain poorly understood. In this study, we used LC-MS/MS technology to compare the peptide profiling between pancreatic tissue of T1DM mice and pancreatic tissue of matched control groups. A total of 106 peptides were differentially expressed in T1DM pancreatic tissue, including 43 upregulated and 63 downregulated peptides. Most of the precursor proteins are insulin. Further bioinformatics analysis (GO and pathway analysis) indicated that the potential functions of these differential peptides were tightly related to regulation of endoplasmic reticulum stress. In conclusion, this study highlights new candidate peptides and provides a new perspective for exploring T1DM pathogenesis and clinical treatments.

## 1. Introduction

Type 1 diabetes mellitus (T1DM) is a common autoimmune disease characterized by the apoptosis of *β* cells and absolute deficiency of insulin secretion, which leads to chronic hyperglycemia, polydipsia, ketoacidosis, and other metabolic disorder [1, 2]. In recent years, the global incidence of T1DM has increased rapidly. It is estimated to account for 5-10% of all diabetes cases worldwide and is expected to surge by 3% per year [3]. The etiology and pathogenesis of T1DM are complex and have not been fully elucidated up to now. At present, the main clinical treatment is insulin injection, while insulin dose, pain of injection, anxiety, and social acceptability bringing great physical and mental trauma to the patients [4]. Therefore, it is urgent to further understand the pathogenesis of T1DM and explore effective treatment approaches.

Endogenous peptides are bioactive substances that can regulate many physiological and pathological processes, including anti-infection, metabolic regulation, antioxidant, and immune regulation [5, 6]. In recent years, a variety of bioactive peptides have been successfully isolated and identified from human, animal, and plant. For instance, cathelicidin is an antimicrobial peptide that not only protects against islet inflammation but also alleviates autoimmune diseases [7, 8]. Amylin, a bioactive peptide, is present in pancreas cell and plays a crucial role in regulating blood sugar [9]. In addition, glucagon-like peptide 1 (GLP-1) receptor agonists such as Lilarutin have been used as drugs for the treatment of diabetes [10]. These studies stimulated our interest in the study of bioactive peptides associated with diabetes. However, the function of the bioactive peptides in T1DM pathogenesis has rarely been researched.

Herein, liquid chromatography tandem mass spectrometry (LC-MS/MS) was employed to compare bioactive peptides in different pancreatic tissue from control and T1DM model mice. The bioinformatics characteristics and cleavage sites of these differentially expressed peptides were also analyzed. Furthermore, we characterized these differentially expressed peptides using Gene Ontology (GO) and pathway analyses and explored potential peptides associated with T1DM. The data suggested that the altered peptidomic profiles in the pancreatic tissues may play an important role in T1DM development.

## 2. Methods

### 2.1. Animal Model

Wild-type C57BL/6J mice (male, 6 weeks old) were purchased from the Experimental Animal Center of Nantong University. All mice were housed in a specific-pathogen free room with a 12 h light-dark cycle and adequate chow and water. The animals were randomly divided into two groups: T1DM group and control group. The mice of the T1DM group were fasted overnight and then intraperitoneally injected with STZ (Sigma-Aldrich) at a dose of 45 mg/kg body weight for 5 consecutive days, whereas the mice of control group received the same volume of citrate acid-sodium citrate buffer (pH 4.54). Mice with random blood glucose levels greater than 16.7 mM were considered diabetic. This experiment was approved by the Animal Ethics Committee of the Affiliated Maternity and Child Health Care Hospital of Nantong University in accordance with the National Institutes of Health Guide for the Care and Use of Laboratory Animals.

### 2.2. Peptide Extraction

Each collected pancreatic tissue was washed with precooled PBS, then ground into powder in liquid nitrogen, and finally mixed with a four times volume of lysate (PBS containing 1 mM PMSF). The mixture was placed on ice for 5 minutes and then centrifuged at 12,000 g for 10 min at 4°C. The equal volume of 100% ACN (acetonitrile) was added to the supernatant and then centrifuged at 10,000 g for 10 min. Transfer the freeze-dried supernatant to a prewetted 10 kD ultrafiltration tube (Merck Millipore, UFC501096, Germany) and centrifuged at 10,000 g for 20 min at 4°C. The filtrates were collected, and the peptides were desalted using C18 columns. The desalted peptide solution was vacuum dried and immediately frozen at −80°C until MS analysis.

### 2.3. Isobaric Tags for Relative and Absolute Quantitation (iTRAQ)

The peptides were dissolved with 0.5 M TEAB (100 *μ*g), labeled according to manufacturer's instructions of iTRAQ-8 kit (SCIEX), and then, the mixed peptide samples were separated by grade using Ultimate 3000 HPLC system (Thermo DINOEX, USA). The chromatographic column was Durashell C18 column (5 m, 100A, 4.6 × 250 mm). The concentration of ACN gradually increased under alkaline conditions to separate the peptide segment, and the flow rate was 1 ml/min, and one tube was collected every minute. A total of 42 secondary fractions were collected and incorporated into 12 components, which were desalted and vacuum dried on the strata-x column.

### 2.4. LC-MS/MS Analysis

The peptide samples were redissolved in a 2% acetonitrile/0.1% formic acid (FA) solution and analyzed using a TripleTOF 5,600 plus mass spectrometer coupled to an Eksigent NanoLC System (SCIEX, USA). Peptides were loaded onto a C18 trap column (5 *μ*m, 100 *μ*m × 20 mm) and eluted at 300 nl/min onto a C18 analytical column (3 *μ*m, 75 *μ*m × 150 mm) in the gradient as long as 90 min. These two mobile phases included buffer A (2% acetonitrile/0.1% formic acid/98% H_2_O) and buffer B (98% acetonitrile/0.1% formic acid/2% H_2_O). For information-dependent acquisition (IDA), the first-order mass spectrogram was scanned at 250 ms accumulation time, and the second-order mass spectrogram of 30 precursor ions was collected at 50 ms accumulation time. The collections of MS1 spectra were in the range 350-1500 m/z, and MS2 spectra were in the range of 100-1500 m/z. The dynamic exclusion time of the precursor ions was set to 15 s.

### 2.5. MS/MS File Data Analysis

ProteinpilotTM v4.5 was adopted to analyze the original MS/MS file data. For peptides identification, the Paragon algorithm was employed against the Mus-musculus SwissProt sequence database. We further filtered these data according to the result of proteinpilot. We consider unused score ≥ 1.3 (confidence > 95%) and proteins containing at least one unique peptide per protein as trusted proteins. For the identified peptide and protein quantification, we consider that the credible peptide is used for protein quantification with the credibility of more than 95%.

### 2.6. Bioinformatics Analyses

We analyzed the molecular weight (MW) and isoelectric point (pI) of the peptides by using the online tool ProtParam (http://web.expasy.org/protparam/). The UniProt database (https://www.uniprot.org/peptidesearch/), Pfam (http://pfam.xfam.org/), and STRING (http://string-db.org/) were used to determine the potential function and interaction of peptides and their protein precursors. Gene Ontology (GO) analysis (http://www.geneontology.org), containing cellular component, molecular functions, and biological processes, was used predict the latent functions of the identified peptides. Each peptide and their protein precursors were imported into the Ingenuity Pathway Analysis software v7.1 for pathway analysis and predicting latent biologic, biochemical, and molecular functions of the given proteins, which were incorporated into an associated network map. The online search tool (https://blast.ncbi.nlm.nih.gov/Blast.cgi) was employed for homology comparison. The PyMOL Molecular Graphics System (CA) was established to model the structures of indicated peptides.

### 2.7. Statistical Analysis

The results of the bioinformatics analysis were analyzed using GraphPad Prism 7, and the data were shown as the mean ± standard deviation (SD). Peptides with a fold change larger than 1.2 or <0.83 with a Student's *t*-test *p* value <0.05 were selected as differently expressed peptides.

## 3. Results

### 3.1. Identification of Differentially Expressed Peptides in the Pancreatic Tissue from T1DM Mice

**Figure 1:**
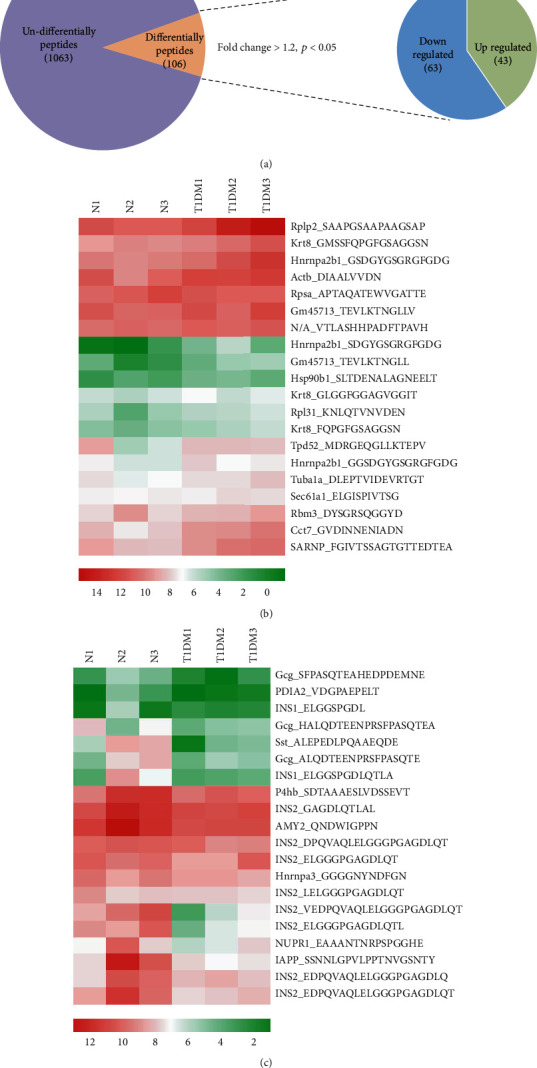
Differently expressed peptides between T1DM and normal mice. (a) Of the 1,063 nonredundant peptides, 106 peptides were significantly differentially expressed in the T1DM mice (fold change ≥ 1.2 and *p* < 0.05), including 43 upregulated and 63 downregulated peptides. Hierarchical clustering showed significant differences in expression between the two groups. The twenty upregulated (b) and downregulated (c) peptides with the highest fold changes were displayed. *N*: control; T1DM: type 1 diabetes mellitus; high expression: red; low expression: green.

Endogenous peptides from the pancreatic tissue of T1DM mice and control groups were directly analyzed using LC-MS/MS after iTRAQ labeling. A total of 1063 nonredundant peptides were identified, among which 106 peptides were differentially expressed in the T1DM mice and control groups (fold change > 1.2, *p* < 0.05), including 43 upregulated peptides and 63 downregulated peptides (Figure 1(a)). All of the differentially expressed peptides are listed in Table S1. The twenty upregulated and downregulated peptides detected in all samples had the highest range of change, which was graphically represented by hierarchical clustering analysis (Figures 1(b) and 1(c)).

### 3.2. Characteristics of Differentially Expressed Peptides

**Figure 2:**
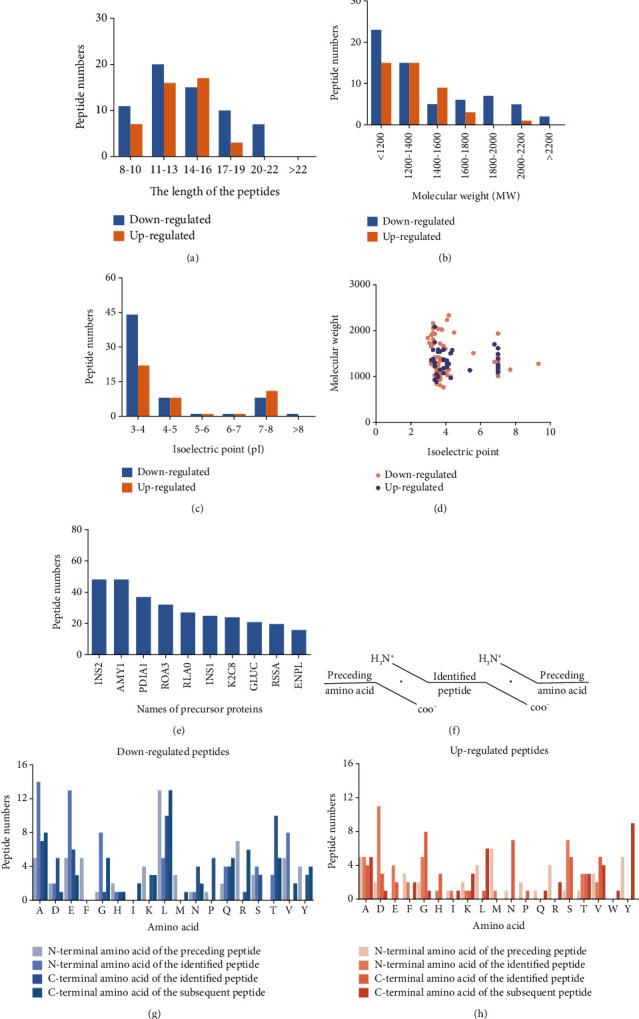
Characteristics of differentially expressed peptides: (a) amino acid of peptides; (b) molecular weight (MW); (c) isoelectric point (pI); (d) scatter plot of MW versus pI; (e) peptides shared the same precursor proteins; (f) cleavage method of peptide; (g) four cleavage sites in downregulated peptides; (h) four cleavage sites in upregulated peptides.

To characterize the general feature of differentially peptides, we further analyzed the relative molecular weight (MW), isoelectric point (pI), amino numbers, and distribution of pI vs. MW. The results shown that the majority of these peptides were 8-16 amino acids in length (Figure 2(a)); the MW varied from 769.9 Da to 2324.5 Da with 75% between 1000 and 1800 Da (Figure 2(b)), and pI varied from 3.0 to 9.3, with 77% between 3 and 5 (Figure 2(c)). Furthermore, the distribution of pI vs. MW could divide these peptides into two groups around pI3 and pI7 (Figure 2(d)). There were several peptides derived from the same precursor protein. The top ten precursor proteins are listed in Figure 2(e), and the largest number of peptides is derived from INS2. Then, UniProt and Pfam databases were used to analyze the functional domains of these peptides. A total of 36 downregulated and 12 upregulated peptides located in the domains of their precursor proteins are listed in Table 1.

### 3.3. Cleavage Site Patterns of Differentially Expressed Peptides

Peptides are mainly cleaved by proteolytic enzyme, and the regulation of their level depends on the type and activity of protease. In other words, by analyzing the characteristic pattern of peptide cleavage sites, the functional differences that may be caused by the enzyme cleavage process can be found. The diagram of the cleavage site distribution is showed in Figure 2(f). Four cleavage sites (C-terminal amino acid of the preceding peptide, C-terminal amino acid of the identified peptide, N-terminal amino acid of the identified peptide, N-terminal amino acid of the identified peptide) were researched for each up- and downregulated peptide (Figures 2(g) and 2(h)). The four dominant cleavage sites in the upregulated peptides were alanine (A), lysine (K), threonine (T), and glycine (G), while in the downregulated peptides were alanine (A), lysine (K), lysine (K), and proline (P).

### 3.4. Gene Ontology (GO) and Pathway Analysis

**Figure 3:**
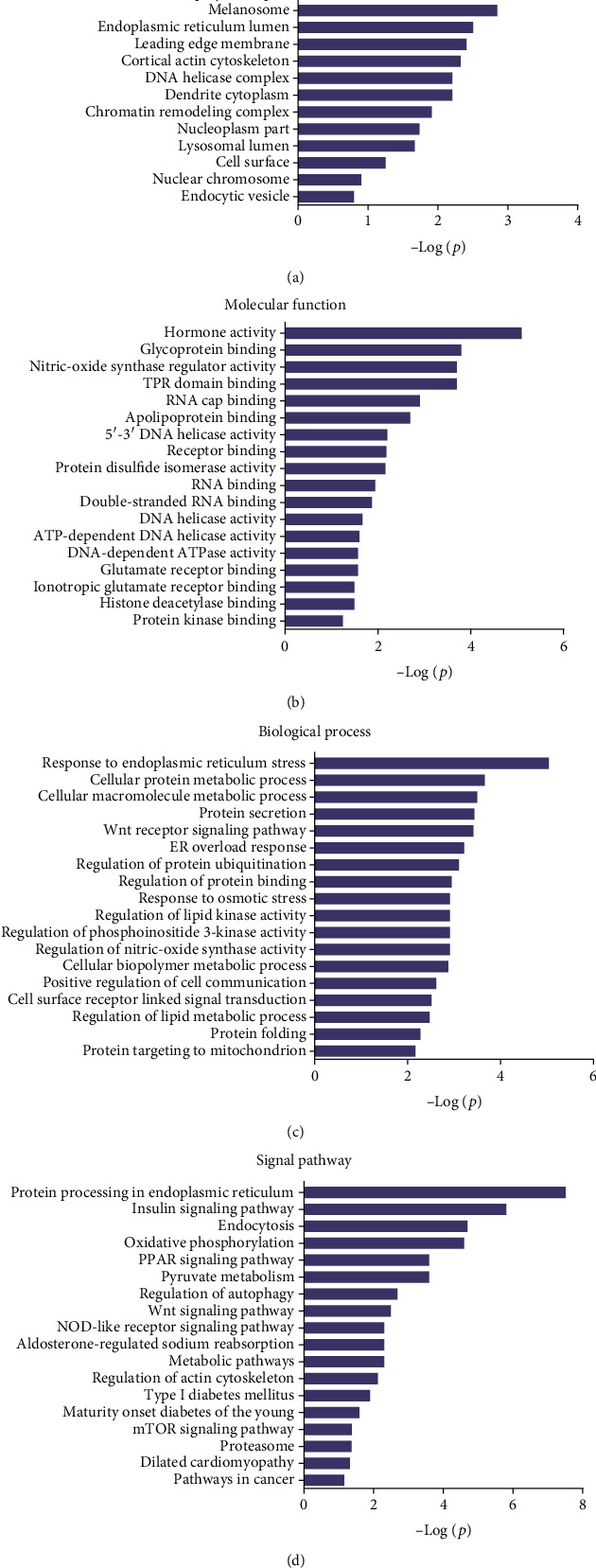
Gene Ontology (GO) and pathway analysis of these precursor proteins: (a) cellular components; (b) molecular functions; (c) biological processes; (d) pathway analysis.

Gene Ontology (GO) and pathway analysis were used to further understand the potential functions of these differential peptides and their corresponding precursor proteins. Cell projection membrane, H4/H2A histone acetyltransferase complex, small nucleolar ribonucleoprotein complex, histone acetyltransferase complex, pigment granule, cell projection part, etc., were the most highly enriched cellular components (Figure 3(a)), whereas hormone activity, glycoprotein binding, nitric-oxide synthase regulator activity, TPR domain binding, RNA cap binding, etc., were the highest enriched molecular functions (Figure 3(b)). Finally, response to endoplasmic reticulum stress, cellular protein metabolic process, cellular macromolecule metabolic process, protein secretion, Wnt receptor signaling pathway, ER overload response, etc., were the most highly enriched biological processes (Figure 3(c)). The pathway analysis revealed that the differentially expressed peptides and their corresponding precursor proteins were involved in protein processing in endoplasmic reticulum, insulin signaling pathway, endocytosis, oxidative phosphorylation, and PPAR signaling pathway, among others (Figure 3(d)).

### 3.5. Functional Clustering by IPA

**Figure 4:**
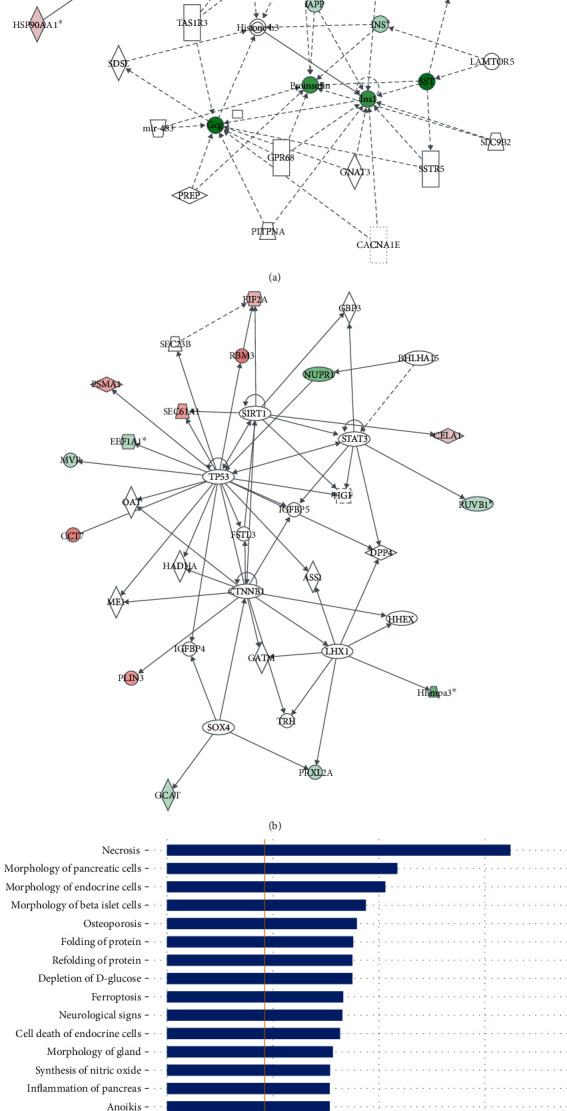
Functional clustering by IPA. (a) Network analysis of “cancer, endocrine system disorders, organismal injury and abnormalities”. (b) Network analysis of “carbohydrate metabolism, cell death and survival, skeletal and muscular disorders” (upregulation, red; downregulation, green). (c) The top twenty precursor proteins related disease function.

The IPA software was employed to produce the interaction network of the precursor proteins of 106 differentially expressed peptides and high-scoring networks that may be linked to the endocrine system. Both “cancer, endocrine system disorders, organismal injury and abnormalities” and “carbohydrate metabolism, cell death and survival, skeletal and muscular disorders” were identified (Figures 4(a) and 4(b)).

The differentially expressed peptides were also entered into the IPA software to conduct a comprehensive analysis of the precursor proteins and their functional analysis of related diseases. The data analysis suggested that some of the precursor proteins were related to endocrine system development and function. These precursor proteins were predicted to have roles in necrosis (*p* = 7.82*E* − 09), morphology of pancreatic cells (*p* = 3.69*E* − 06), morphology of endocrine cells (*p* = 6.93*E* − 06), and morphology of beta islet cells (*p* = 2.02*E* − 05). The top twenty precursor proteins related disease function are listed in Figure 4(c).

## 4. Discussion

Type 1 diabetes mellitus (T1DM) is characterized by the dysfunction and apoptosis of pancreatic *β* cells [11, 12]. It occurs primarily in children and adolescents. Although T1DM is relatively rare, it still increases at an annual rate of 3%. However, the etiology and pathogenesis of T1DM are complex and have not been fully elucidated up to now [13]. Long-term insulin injection and long-term metabolic disorders cause many acute and chronic complications. Recently, some peptide drugs have provided new ideas for the treatment of diabetes. Peptides display a multitude of biological activities such as stabilizing mitochondrial proteins, stimulating glucose uptake, and regulating signal transduction [14, 15]. So, we attempted to synthesize the peptides that may be involved in regulating insulin secretion to provide important insights into the molecular mechanism of pancreatic *β* cell apoptosis.

In our study, we collected pancreas from normal and T1DM model mice for peptide extractions with 10 kDa MWCO filters, which could remove redundant proteins and ensure the purity of the extracted peptides. Then, we employed TMT-labeled analysis to compare the composition of pancreas peptides from the T1DM and control group and identified 1063 peptides from LC-MS/MS. A total of 106 peptides were differentially secreted in pancreas tissues of T1DM mice, of which 43 peptides were upregulated and 63 were downregulated. The MWs of the identified peptides were less than 2.5 kDa, and 75% of the peptides were mainly distributed in the range of 1.0-1.5 kDa. The pI of the peptides ranged from 3 to 5, accounting for 77% of all peptides. This data indicated that the MWCO filter is effective in our process of extracting peptides.

Blood glucose balance regulation is a part of the body life activity regulation and is an important condition to maintain the homeostasis of the body environment. There are many kinds of hormones in human body that can regulate the content of blood glucose, but insulin and glucagon are the main ones [16–18]. In our study, 106 differentially expressed peptides from 57 precursor proteins were identified from different pancreatic tissues. Most of the precursor proteins are insulin. It is quite possible that the peptides upregulated can raise blood sugar, whereas downregulated peptides could be reduced blood sugar. Therefore, we can test the function of these peptides by gene silencing or synthetic peptide treatment experiments. Further study on the function of these differentially expressed peptides will provide a new approach for the treatment of T1DM.

Protein domain is a region with specific sequence and structure that can folding and traveling functions independently. We identified 48 peptides located in function domains, 22 of which were insulin domain. The main role of insulin is to reduce blood sugar; it can increase cell permeability to monosaccharides, amino acids, and fatty acids and is the best treatment for type 1 diabetes so far [19–21]. Thioredoxin domain is a class of small redox proteins known to be present in all organisms. Recent research shows that thioredoxin interacting protein is an important factor in regulating pancreatic *β* cell dysfunction and death and is a key process in the pathogenesis of T1DM [22]. Therefore, the confirmation of these peptides is very important in the future studies.

The biological functions of these peptides were predicted by the analysis of the precursor proteins. GO and pathway analysis showed that the potential functions of these differential peptides were tightly related to regulation of endoplasmic reticulum stress. Recent observations suggest that endoplasmic reticulum stress mediated apoptosis plays an important role in pancreatic *β* cell destruction [23, 24]. The early biosynthesis of insulin is mainly in the endoplasmic reticulum, and the pancreatic *β* cells have a highly active and developed ER [25]. It is one of the most sensitive cells to endoplasmic reticulum stress. Persistent ERS and defective ERS signaling pathways in *β* cells can cause the dysregulation of ER homeostasis, which results in pancreatic *β* cell apoptosis and autoimmune response and then developing into diabetes mellitus [26, 27]. These results indicated that these peptides participate are involved in dysfunction and death of pancreatic *β* cells and play an important regulatory role.

In summary, using LC-MS/MS technology, we have identified the peptide profiles of pancreatic tissues from T1DM mice and matched normal mice. Our results suggest that some peptides may participate in pancreatic dysfunction and diabetes. The emergence of these peptides provides a new perspective for exploring T1DM pathogenesis and clinical treatments. Furthermore, the detail mechanism of these endogenous peptides to regulate pancreatic *β* cells needs further study.

## Figures and Tables

**Table 1:** Differentially expressed peptides located in function domain based on Uniport and Pfam database.

Protein	Peptide sequence	Location	Domain location	Domain description
Downregulated peptides				
PDIA2	VDGPAEPELT	103-112	29-155	Thioredoxin 1
INS1	ELGGSPGDL	67-75	28-107	Insulin
INS1	ELGGSPGDLQTLA	67-79	28-107	Insulin
INS2	GAGDLQTLAL	73-82	28-109	Insulin
AMY2	QNDWIGPPN	366-374	26-410	Aamy
INS2	DPQVAQLELGGGPGAGDLQT	60-79	28-109	Insulin
INS2	ELGGGPGAGDLQT	67-79	28-109	Insulin
INS2	LELGGGPGAGDLQT	66-79	28-109	Insulin
INS2	VEDPQVAQLELGGGPGAGDLQT	58-79	28-109	Insulin
INS2	ELGGGPGAGDLQTL	67-80	28-109	Insulin
NUPR1	EAAANTNRPSPGGHE	49-63	21-77	Phospho-p8
IAPP	SSNNLGPVLPPTNVGSNTY	56-74	29-79	Calcitonin
INS2	EDPQVAQLELGGGPGAGDLQ	59-78	28-109	Insulin
INS2	EDPQVAQLELGGGPGAGDLQT	59-79	28-109	Insulin
INS2	VEDPQVAQLELGGGPGAGDLQ	58-78	28-109	Insulin
INS2	ELGGGPGAGDLQ	67-78	28-109	Insulin
INS1	EVEDPQVEQLELGGSPGD	57-74	28-107	Insulin
Hbbt1	LLVVYPWTQR	36-45	2-251	Globin
INS2	VEDPQVAQLELGGGPGAGD	58-76	28-109	Insulin
INS2	EDPQVAQLELGGGP	59-72	28-109	Insulin
INS2	QLELGGGP	65-72	28-109	Insulin
INS2	EDPQVAQLELGGGPGAGD	59-76	28-109	Insulin
INS2	GGPGAGDLQTL	70-80	28-109	Insulin
Eif4a1	NTSIEEMPLN	392-401	245-406	Helicase C-terminal
UB2L3	VNDPQPEHPL	112-121	2-149	UBC core
Rplp0	ADPSAFAAAAPA	275-281	231-316	Ribosomal-60s
INS2	QLELGGGPGAGDL	65-77	28-109	Insulin
INS2	LGGGPGAGDL	68-77	28-109	Insulin
INS2	GGGPGAGDLQT	69-79	28-109	Insulin
Actb	VAPEEHPVLLTEAPLNPK	96-113	2-375	Actin
IAPP	GPVLPPTNVGSNTY	61-74	29-79	Calcitonin
INS2	ELGGGPGAGDL	67-77	28-109	Insulin
AMY2	LNPNNREFPAVP	134-145	26-410	Aamy
Ruvbl1	AQTEGINISEEALNHLGEIGTK	379-400	374-439	TIP49-c
MDH1	GVISDGNSYGVPDD	270-283	156-331	Ldh_1_N
B2m	AHTEFTPTETDT	86-97	25-114	Ig-like
Upregulated peptides				
Actb	DIAALVVDN	4-12	2-375	Actin
Rpsa	APTAQATEWVGATTE	279-293	202-295	40S_SA_C
Hsp90b1	SLTDENALAGNEELT	119-133	96-255	HATPase-c
Tuba1a	DLEPTVIDEVRTGT	69-82	3-214	Tubulin
Rbm3	DYSGRSQGGYD	131-141	81-154	Disordered
Cct7	GVDINNENIADN	476-487	32-524	Cpn60_TCP1
ERP27	DLEIPIVS	66-73	39-152	Thioredoxin
UBN2	DETDPFIDNSEA	196-207	180-231	HUN
Ptma	SDAAVDTSSEITTKD	62-76	62-170	Prothymosin
AMY2	SAEDPFIAIHADS	494-506	419-507	Aamy_C
Atp5f1b	EVAQHLGES	98-106	63-129	ATP-synt_ab_N
P4hb	DRTVIDYNGE	453-462	348-477	Thioredoxin

## Data Availability

The data used to support the findings of this study are included within the article.

## Supplementary Materials

Supplementary Materials Table S1: a total of 106 peptides were differentially expressed in the T1DM mice and control groups (fold change > 1.2, *p* < 0.05), including 43 upregulated peptides and 63 downregulated peptides.
