# Supplementary material for: Peptidome Analysis of Pancreatic Tissue Derived from T1DM Mice: Insights into the Pathogenesis and Clinical Treatments of T1DM
Source: Biomed Res Int. 2021 May 21;2021:9987042. doi: 10.1155/2021/9987042 (PMC8164536; doi:10.1155/2021/9987042)
Supplement: Supplementary Materials — Table S1: a total of 106 peptides were differentially expressed in the T1DM mice and control groups (fold change > 1.2, p < 0.05), including 43 upregulated peptides and 63 downregulated peptides. [file 9987042.f1.docx]

Table 1 Differentially expressed peptides in the pancreatic tissue of TIDM mouse model

| **Accession** | **Gene** | **Protein** | **Peptide** | **MW (KDa)** | **Fold change** | ***p*-value** |
| --- | --- | --- | --- | --- | --- | --- |
| Down-regulated peptides | | | | | | |
| P55095 | Gcg | Glucagon | SFPASQTEAHEDPDEMNE | 2337.001 | 42.55319 | 0.012103 |
| D3Z6P0 | PDIA2 | Protein disulfide-isomerase A2 | VDGPAEPELT | 1330.692 | 28.6533 | 0.007842 |
| P01325 | INS1 | Insulin-1 | ELGGSPGDL | 1147.603 | 26.95418 | 0.025672 |
| P55095 | Gcg | Glucagon | HALQDTEENPRSFPASQTEA | 2531.219 | 23.14815 | 0.002622 |
| P60041 | Sst | Somatostatin | ALEPEDLPQAAEQDE | 1957.942 | 20.61856 | 0.034927 |
| P55095 | Gcg | Glucagon | ALQDTEENPRSFPASQTE | 2323.124 | 20.3666 | 0.018279 |
| P01325 | INS1 | Insulin-1 | ELGGSPGDLQTLA | 1560.83 | 20.04008 | 0.010481 |
| Q3TGS0 | P4hb | Protein disulfide-isomerase | SDTAAAESLVDSSEVT | 1884.911 | 17.45201 | 0.025399 |
| P01326 | INS2 | Insulin-2 | GAGDLQTLAL | 1261.719 | 13.73626 | 0.041385 |
| Q8C5B4 | AMY2 | Amylase alpha 2A | QNDWIGPPN | 1343.678 | 12.87001 | 0.025399 |
| P01326 | INS2 | Insulin-2 | DPQVAQLELGGGPGAGDLQT | 2226.144 | 12.18027 | 0.027903 |
| P01326 | INS2 | Insulin-2 | ELGGGPGAGDLQT | 1474.757 | 9.191176 | 0.008433 |
| Q8BG05 | Hnrnpa3 | Heterogeneous nuclear ribonucleoprotein A3 | GGGGNYNDFGN | 1374.611 | 7.686395 | 0.045137 |
| P01326 | INS2 | Insulin-2 | LELGGGPGAGDLQT | 1587.841 | 7.204611 | 0.031402 |
| P01326 | INS2 | Insulin-2 | VEDPQVAQLELGGGPGAGDLQT | 2454.254 | 7.137759 | 0.02668 |
| P01326 | INS2 | Insulin-2 | ELGGGPGAGDLQTL | 1587.841 | 7.017544 | 0.007022 |
| Q9WTK0 | NUPR1 | Nuclear protein 1 | EAAANTNRPSPGGHE | 1810.886 | 6.98812 | 0.04248 |
| P12968 | IAPP | Islet amyloid polypeptide | SSNNLGPVLPPTNVGSNTY | 2233.165 | 6.613757 | 0.025672 |
| P01326 | INS2 | Insulin-2 | EDPQVAQLELGGGPGAGDLQ | 2254.138 | 6.430868 | 0.025021 |
| P01326 | INS2 | Insulin-2 | EDPQVAQLELGGGPGAGDLQT | 2355.186 | 5.927682 | 0.008433 |
| A9JSM3 | Tmem238 | Transmembrane protein 238 | TLEAGSVAAGTGSE | 1552.789 | 5.128205 | 0.043755 |
| P11679 | Krt8 | Keratin type II cytoskeletal 8 | QPGFGSAGGSNTFS | 1616.774 | 4.975124 | 0.025672 |
| P01326 | INS2 | Insulin-2 | VEDPQVAQLELGGGPGAGDLQ | 2353.207 | 4.714757 | 0.025399 |
| Q3TGS0 | P4hb | Protein disulfide-isomerase | LESGGQDGAGDDEDLDLE | 2137.944 | 4.675082 | 0.034927 |
| P01326 | INS2 | Insulin-2 | ELGGGPGAGDLQ | 1373.709 | 4.43459 | 0.007842 |
| P01325 | INS1 | Insulin-1 | EVEDPQVEQLELGGSPGD | 2201.064 | 4.264392 | 0.037501 |
| A8DUK2 | Hbbt1 | Beta-globin | LLVVYPWTQR | 1577.924 | 3.984064 | 0.020044 |
| P01326 | INS2 | Insulin-2 | VEDPQVAQLELGGGPGAGD | 2112.064 | 3.968254 | 0.024896 |
| P01326 | INS2 | Insulin-2 | EDPQVAQLELGGGP | 1712.889 | 3.856537 | 0.010559 |
| P01326 | INS2 | Insulin-2 | QLELGGGP | 1074.586 | 3.810976 | 0.011662 |
| P99027 | Rplp2 | 60S acidic ribosomal protein P2 | AAPGSAAPAAGSAP | 1398.741 | 3.49895 | 0.025672 |
| P01326 | INS2 | Insulin-2 | EDPQVAQLELGGGPGAGD | 2012.996 | 3.495281 | 0.013588 |
| P01326 | INS2 | Insulin-2 | GGPGAGDLQTL | 1288.693 | 3.439972 | 0.003704 |
| D3Z6P0 | PDIA2 | Protein disulfide-isomerase A2 | AEEAPTLRL | 1302.745 | 3.330003 | 0.048631 |
| Q3TGK7 | Eif4a1 | RNA helicase | NTSIEEMPLN | 1450.728 | 3.290556 | 0.048922 |
| P68037 | UB2L3 | Ubiquitin-conjugating enzyme E2 L3 | VNDPQPEHPL | 1448.757 | 3.171583 | 0.008433 |
| P14869 | Rplp0 | 60S acidic ribosomal protein P0 | ADPSAFAAAAPA | 1362.709 | 3.164557 | 0.045838 |
| P55095 | Gcg | Glucagon | ALQDTEENPRSFPAS | 1964.975 | 2.977077 | 0.044201 |
| O88986 | Gcat | Glycine C-acetyltransferase | VEVGRLHGALP | 1450.856 | 2.96472 | 0.02668 |
| P01326 | INS2 | Insulin-2 | QLELGGGPGAGDL | 1486.793 | 2.920561 | 0.024896 |
| Q91X79 | CELA1 | Chymotrypsin-like elastase family member 1 | TEDVPETDARVVGGAEA | 2019.006 | 2.868617 | 0.018279 |
| P01326 | INS2 | Insulin-2 | LGGGPGAGDL | 1116.608 | 2.830456 | 0.025672 |
| Q3U125 | Prxl2a | Peroxiredoxin-like 2A | AALEYLEDIDLK | 2000.129 | 2.796421 | 0.010559 |
| P01326 | INS2 | Insulin-2 | GGGPGAGDLQT | 1232.63 | 2.649709 | 0.010466 |
| P10126 | EF1A1 | Elongation factor 1-alpha | ESFSDYPPLG | 1414.692 | 2.622607 | 0.024896 |
| Q8C2S9 | Mvp | Major vault protein | GLLGLGSDGQPPVQ | 1640.904 | 2.603489 | 0.010559 |
| Q3UAA9 | Actb | Actin beta | VAPEEHPVLLTEAPLNPK | 2561.468 | 2.53357 | 0.010481 |
| D3Z6P0 | PDIA2 | Protein disulfide-isomerase A2 | VDVAADNSHVL | 1442.767 | 2.477701 | 0.025672 |
| P08003 | PDIA4 | Protein disulfide-isomerase A4 | AMEPEEFDSDT | 1573.676 | 2.416626 | 0.025399 |
| P12968 | IAPP | Islet amyloid polypeptide OS | GPVLPPTNVGSNTY | 1718.915 | 2.359047 | 0.037147 |
| P01326 | INS2 | Insulin-2 | ELGGGPGAGDL | 1245.651 | 2.352941 | 7.60E-05 |
| P11499 | Hsp90ab1 | Heat shock protein HSP 90-beta | AAVPDEIPPLEGDEDAS | 2027.984 | 2.212389 | 0.031548 |
| Q921T2 | TOR1AIP1 | Torsin-1A-interacting protein 1 | AGERWQAEGPGEGWA | 1641.717 | 2.206531 | 0.025302 |
| Q9CY06 | N/A | GLOBIN domain-containing protein | SHHPADFTPAVH | 1314.61 | 2.19106 | 0.004476 |
| Q91V38 | Hsp90b1 | Heat shock protein 90 beta family member 1 | TETVEEPLEED | 1593.756 | 2.148689 | 0.049958 |
| Q61177 | Csnk2a1 | Casein kinase II alpha subunit | AANSLGIPVPA | 1312.766 | 2.123593 | 0.030342 |
| Q8C5B4 | AMY2 | Amylase alpha 2A | LNPNNREFPAVP | 1670.905 | 1.998002 | 0.012837 |
| Q9CY06 | N/A | GLOBIN domain-containing protein | AAGHLDDLPGA | 1339.704 | 1.95427 | 0.037501 |
| P60122 | Ruvbl1 | RuvB-like 1 | AQTEGINISEEALNHLGEIGTK | 2931.576 | 1.917178 | 0.037501 |
| P99027 | Rplp2 | 60S acidic ribosomal protein P2 | DSVGIEADDDRLN | 1721.838 | 1.871958 | 0.028648 |
| P14152 | MDH1 | Malate dehydrogenase 1 | GVISDGNSYGVPDD | 1697.805 | 1.732502 | 0.018279 |
| O88569 | Hnrnpa2b1 | Heterogeneous nuclear ribonucleoproteins A2/B1 | GGGNYGPGGSGGSGGY | 1603.717 | 1.717623 | 0.034927 |
| Q9D239 | B2m | Beta-2-microglobulin | AHTEFTPTETDT | 1652.784 | 1.362583 | 0.044659 |
| Up-regulated peptides | | | | | | |
| P99027 | Rplp2 | 60S acidic ribosomal protein P2 | SAAPGSAAPAAGSAP | 1485.773071 | 18.275 | 0.01459452 |
| P11679 | Krt8 | Keratin type II cytoskeletal 8 | GMSSFQPGFGSAGGSN | 1790.820068 | 17.9816 | 0.02515991 |
| O88569 | Hnrnpa2b1 | Heterogeneous nuclear ribonucleoproteins A2/B1 | GSDGYGSGRGFGDG | 1591.716919 | 11.9242 | 0.04247967 |
| Q3UAA9 | Actb | Actin beta | DIAALVVDN | 1232.691895 | 11.0017 | 0.04076326 |
| P14206 | Rpsa | 40S ribosomal protein SA | APTAQATEWVGATTE | 1835.920776 | 7.624 | 0.02266543 |
| A0A1B0GS68 | Gm45713 | 60S ribosomal protein L13a | TEVLKTNGLLV | 1794.107666 | 6.7076 | 0.01827859 |
| Q9CY06 | N/A | GLOBIN domain-containing protein | VTLASHHPADFTPAVH | 2003.053223 | 6.5277 | 0.01080182 |
| O88569 | Hnrnpa2b1 | Heterogeneous nuclear ribonucleoproteins A2/B1 | SDGYGSGRGFGDG | 1534.695557 | 6.013 | 0.01827859 |
| A0A1B0GS68 | Gm45713 | 60S ribosomal protein L13a | TEVLKTNGLL | 1695.039185 | 5.6355 | 0.02934318 |
| Q91V38 | Hsp90b1 | Heat shock protein 90 beta family member 1 | SLTDENALAGNEELT | 1879.931763 | 5.5557 | 0.02567162 |
| P11679 | Krt8 | Keratin type II cytoskeletal 8 | GLGGFGGAGVGGIT | 1422.777344 | 5.1863 | 0.03295298 |
| P62900 | Rpl31 | 60S ribosomal protein L31 | KNLQTVNVDEN | 1881.041748 | 5.0451 | 0.01827859 |
| P11679 | Krt8 | Keratin type II cytoskeletal 8 | FQPGFGSAGGSN | 1428.694092 | 4.249 | 0.01827859 |
| E9PUA7 | Tpd52 | Tumor protein D52 | MDRGEQGLLKTEPV | 1918.013672 | 4.0925 | 0.04984269 |
| O88569 | Hnrnpa2b1 | Heterogeneous nuclear ribonucleoproteins A2/B1 | GGSDGYGSGRGFGDG | 1648.738403 | 3.7855 | 0.02254707 |
| P68369 | Tuba1a | Tubulin alpha-1A chain | DLEPTVIDEVRTGT | 1847.978271 | 3.5298 | 0.02644371 |
| Q3TJD0 | Sec61a1 | Plug_translocon domain-containing protein | ELGISPIVTSG | 1375.786499 | 3.2475 | 0.01468058 |
| Q8BG13 | Rbm3 | RNA-binding protein 3 | DYSGRSQGGYD | 1507.68457 | 3.0813 | 0.01210332 |
| Q8C5Q5 | Cct7 | T-complex protein 1 subunit eta | GVDINNENIADN | 1590.779175 | 2.866 | 0.0044764 |
| Q9D1J3 | SARNP | SAP domain-containing ribonucleoprotein | FGIVTSSAGTGTTEDTEA | 2046.98999 | 2.6056 | 0.02539938 |
| Q80U89 | mKIAA0034 | MKIAA0034 protein | TAGPSVAVPPQAPFGYG | 1919.009521 | 2.5367 | 0.0385883 |
| Q9CX22 | Cfl1 | Cofilin 1 | ASGVAVSDGVIKV | 1546.887329 | 2.5035 | 0.0122039 |
| Q9D8U3 | ERP27 | Endoplasmic reticulum resident protein | DLEIPIVS | 1188.690918 | 2.4925 | 0.02668024 |
| Q80WC1 | UBN2 | Ubinuclein-2 | DETDPFIDNSEA | 1655.746948 | 2.4484 | 0.02567162 |
| Q8CDZ2 | MT21E | Protein-lysine methyltransferase METTL21E | DDDDDKQVV | 1655.846313 | 2.3542 | 0.04995818 |
| Q3U8X6 | Ptma | Prothymosin alpha | SDAAVDTSSEITTKD | 1884.910645 | 2.287 | 0.00843321 |
| Q9DBG5 | Plin3 | Perilipin-3 | VGPFAPGITEKTPEG | 2107.17749 | 2.2706 | 0.03750086 |
| O88569 | Hnrnpa2b1 | Heterogeneous nuclear ribonucleoproteins A2/B1 | DNYGGGNYGSGS | 1450.626709 | 2.1603 | 0.04334807 |
| Q91X79 | CELA1 | Chymotrypsin-like elastase family member 1 | ISWMNNVIASN | 1567.797119 | 2.1494 | 0.01435078 |
| Q9CX22 | Cfl1 | Cofilin 1 | ASGVAVSDGVIK | 1447.81897 | 1.9897 | 0.03750086 |
| P35564 | Canx | Calnexin | HDGHDDDAIDIEDD | 1884.791626 | 1.9618 | 0.02619998 |
| Q8BTU5 | Psma1 | Proteasome subunit alpha type | AEEPAEKADEPMEH | 2190.07251 | 1.9552 | 0.02567162 |
| Q8C5B4 | AMY2 | Alpha amylase 2A | SAEDPFIAIHADS | 1675.83606 | 1.887 | 0.02298698 |
| Q8BQW4 | Arhgap1 | Rho GTPase activating protein 1 | DLTLDDTSQALN | 1608.814941 | 1.8596 | 0.02539938 |
| Q91XB7 | YIF1A | Yip1 interacting factor homolog A | DPPPLFDDTSGG | 1520.730103 | 1.7723 | 0.01048059 |
| P08003 | PDIA4 | Protein disulfide-isomerase A4 | DLGLSESGEDVNAA | 1679.815674 | 1.6371 | 0.01957123 |
| Q8BJW6 | EIF2A | Eukaryotic translation initiation factor 2A | APSTPLLTVRGSEG | 1687.941162 | 1.599 | 0.04059934 |
| Q3TJD0 | Sec61a1 | Plug_translocon domain-containing protein | EQSEVGSMGA | 1297.612671 | 1.5024 | 0.03955743 |
| P49817 | CAV1 | Caveolin-1 | SGGKYVDSEGH | 1480.710083 | 1.4424 | 0.03492746 |
| Q6ZQ58 | LARP1 | La-related protein 1 | SISSSPSEGTPAVGS | 1665.836426 | 1.432 | 0.01827859 |
| P56480 | Atp5f1b | ATP synthase subunit beta | EVAQHLGES | 1272.661743 | 1.4068 | 0.01856316 |
| Q3TGS0 | P4hb | Protein disulfide-isomerase | DRTVIDYNGE | 1484.741333 | 1.3636 | 0.01116063 |
| P07901 | Hsp90aa1 | Heat shock protein HSP 90-alpha | EEMPPLEGDDDTSRMEEV | 2398.045898 | 1.2106 | 0.03575434 |
